# Association of Arterial PaCO_2_ with the Survival of Mechanically Ventilated Patients with Acute Respiratory Failure: A Multicenter Retrospective Cohort Study

**DOI:** 10.3390/diagnostics16030489

**Published:** 2026-02-05

**Authors:** Lei Chang, Ling Jia, Yue Xu, Yali Qian, Shaodong Zhao, Yanqun Sun, Xuhua Ge, Hongjun Miao

**Affiliations:** 1 Department of Pediatric Intensive Care Unit, Children’s Hospital of Nanjing Medical University, Nanjing 210008, China; leichang2022@outlook.com (L.C.); yanq_sun@163.com (Y.Q.); zhaoshaodong@aliyun.com (S.Z.); sunyanqun@126.com (Y.S.); 2 The Second Affiliated Hospital of Nanjing Medical University, Nanjing 210011, China; 3 Department of Critical Care Medicine, Sir Run Run Hospital, Nanjing Medical University, Nanjing 211100, China; jling98@njmu.edu.cn; 4 Pediatrics Department, Tongzhou District Hospital of Nantong City, Nantong 226300, China; xyxiaocangshu@outlook.com

**Keywords:** arterial partial pressure of carbon dioxide (PaCO_2_), MIMIC-IV, eICU Collaborative Research Database (eICU-CRD), acute respiratory failure, mechanical ventilation

## Abstract

**Background/Objectives**: Acute respiratory failure (ARF) is associated with a high mortality. This study aimed to explore the association of arterial partial pressure of carbon dioxide (PaCO_2_) in relation to survival outcomes in mechanically ventilated patients with ARF. **Methods**: This multicenter retrospective cohort study integrated the data from the eICU Collaborative Research Database (eICU-CRD; n = 10,946), the Medical Information Mart for Intensive Care IV (MIMIC-IV; n = 6683), and clinical records from two university-affiliated intensive care units in China (n = 410). The patients were categorized into low, normal, and high PaCO_2_ groups using a restricted cubic spline model to explore the relationship between PaCO_2_ and mortality. The 28-day survival distributions among the three groups were compared using Kaplan–Meier curves, with statistical significance assessed via the log-rank test. A multivariable Cox proportional hazards model was constructed to evaluate the independent prognostic value of PaCO_2_ for multiple complications. Hazard ratios (HRs) and 95% confidence intervals (CIs) were calculated for the low and high PaCO_2_ groups relative to the normal PaCO_2_ group. **Results**: A U-shaped relationship was observed between PaCO_2_ and mortality, with both low PaCO_2_ (<36.4 mmHg) and high PaCO_2_ (>57.9 mmHg) associated with an increased mortality risk. Kaplan–Meier survival analysis demonstrated that patients in the intermediate PaCO_2_ range (36.4–57.9 mmHg) exhibited the highest survival rate (65.2%), whereas those in the low and high PaCO_2_ groups had significantly lower survival rates (60.0% and 63.2%) (log-rank test, *p* < 0.001). Adjusted survival analyses further revealed that complications such as sepsis and chronic kidney disease significantly influenced the mortality across PaCO_2_ strata. Compared with the intermediate PaCO_2_ group, the hazard of death increased by 25.5% in the low PaCO_2_ group and by 18.9% in the high PaCO_2_ group. **Conclusions**: This retrospective analysis indicates that arterial PaCO_2_ levels within the optimal range are associated with improved survival in patients with acute respiratory failure (ARF) on mechanical ventilation, but prospective studies are needed to establish causality and consider potential confounding factors.

## 1. Introduction

Acute respiratory failure (ARF) is a critical condition marked by the sudden impairment of gas exchange, and responsible for hypoxemia, hypercapnia, or both. Due to its high prevalence and mortality rates, ARF imposes a considerable burden on patients and healthcare systems alike, often requiring mechanical ventilation or treatments at intensive care unit (ICUs) [1,2]. Conventional therapies, such as mechanical ventilation and pharmacological interventions, just obtain limited survival outcomes [3], underscoring the urgent need for more effective treatment strategies.

Recent studies have highlighted the prognostic significance of specific clinical parameters in ARF. For instance, arterial blood gas parameters, particularly the arterial partial pressure of carbon dioxide (PaCO_2_), have shown substantial influences on the mortality [4]. Prior studies have reported that both low and high extremes of PaCO_2_ are associated with worse outcomes in ARF patients, particularly those receiving mechanical ventilation [4,5]. However, previous studies mainly focused on the impact of ventilation modes and intervention timing on survival rates or emphasized the correlation between dynamic changes in PaCO_2_ and prognosis. This study aims to optimize ventilation strategies by setting stable target values for PaCO_2_ to improve patient outcomes. There is currently no clear consensus on the optimal PaCO_2_ target for mechanically ventilated patients. Determining the ideal range for PaCO_2_ can provide valuable reference values for clinicians when implementing lung-protective ventilation.

Here, we performed a comprehensive retrospective analysis utilizing large-scale, multicenter critical care databases, specifically the eICU Collaborative Research Database (eICU-CRD) and the Medical Information Mart for Intensive Care IV (MIMIC-IV). The big clinical data in these databases ensured the generalizability of our findings [6,7]. By evaluating the association between varying levels of PaCO_2_ and survival outcomes in patients with ARF, we provided robust evidence that could inform clinical decision-making and optimize treatment strategies.

## 2. Materials and Methods

### 2.1. Data Sources

This retrospective study drew upon two publicly available, large-scale intensive care databases: the eICU Collaborative Research Database version 2.0 (eICU-CRD 2.0) and the Medical Information Mart for Intensive Care version IV 2.0 (MIMIC-IV 2.0). Additionally, data were obtained from Sir Run Run Hospital (SRRH) and the Second Affiliated Hospital of Nanjing Medical University (SFH, NJUM). The eICU-CRD 2.0, developed by the Philips eICU Research Institute, is a multicenter database covering over 200 hospitals across the United States from 2014 to 2015. This telehealth-oriented database focuses exclusively on adult ICU patients and includes continuous and intermittent vital signs, laboratory measurements, pharmaceutical records, detailed care plan information, admission diagnoses, and treatment information [8]. MIMIC-IV 2.0 is a publicly accessible database containing the medical data of adult patients (aged ≥18 years) admitted to the Beth Israel Deaconess Medical Center in Boston, Massachusetts, USA between 2008 and 2019 involving the demographic data, vital sign measurements, laboratory test results, medication and procedure records, ICD codes, and length of hospital stay [9]. The database has completed data desensitization so that the researchers can use the data without patients’ consent. Anyone who has passed the Collaborative Institutional Training Initiative Program can request access to the database [10]. To obtain access, we passed the online training courses and exams.

### 2.2. Participants

**Figure 1:**
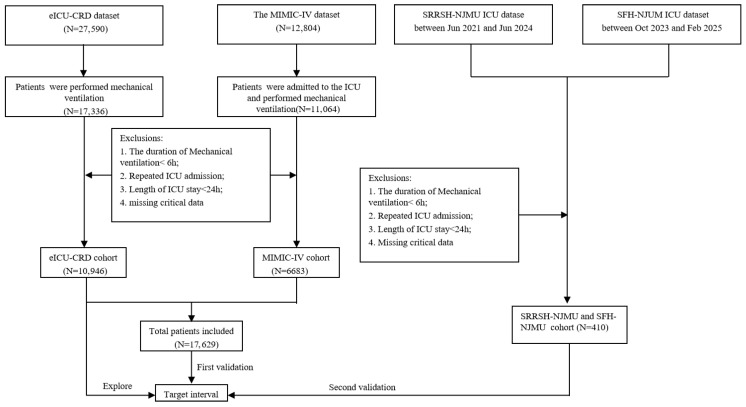
The flow chart of participant selection.

The study population was derived from the eICU-CRD, MIMIC-IV, SRRSH-NJMU, and SFH-NJMU databases and included patients diagnosed with ARF and receiving mechanical ventilation. Diagnoses were set based on the International Classification of Diseases, 9th and 10th Revisions (ICD-9 and ICD-10) by the World Health Organization. Patients were excluded if they met any of the following criteria: (1) duration of mechanical ventilation <6 h; (2) repeated ICU admissions; (3) ICU stay <24 h; or (4) missing key clinical data. The patient selection process is illustrated in Figure 1.

### 2.3. Data Extraction

Clinical data were extracted from the eICU-CRD 2.0 and MIMIC-IV 2.0 databases using structured query language (SQL), and additional data were collected from SRRSH-NJMU and SFH-NJMU. Data within the initial 72 h following ICU admission were collected, and categorized as follows: (1) laboratory tests, including platelet count, white blood cell (WBC) count, hemoglobin concentration, total bilirubin (TBIL), and serum creatinine levels; (2) demographics and vital signs, including sex, age, race, heart rate, respiratory rate, mean arterial pressure (MAP), Sequential Organ Failure Assessment (SOFA) score, Acute Physiology and Chronic Health Evaluation III (APS III) score, and Charlson Comorbidity Index (CCI), as well as ICU-related variables (length of ICU stay and duration of mechanical ventilation); (3) comorbidities, including sepsis, myocardial infarction, congestive heart failure, cerebrovascular disease, chronic lung disease, kidney disease, and severe liver disease. For arterial blood gas analysis, the maximum PaCO_2_ value within 48 h after the initiation of mechanical ventilation was extracted. Respiratory parameters, including respiratory frequency (RF), positive end-expiratory pressure (PEEP), tidal volume (TV), and the PaO_2_/FiO_2_ ratio (P/F ratio), were averaged over the first 72 h of mechanical ventilation. The primary exposure was the time-weighted averages PaCO_2_ (TWA-PaCO_2_) during mechanical ventilation in each patient. The specific calculation method was as follows: All PaCO_2_ measurements were extracted during the patient’s mechanical ventilation time. We arranged them in time order on the time axis, then used the computer to make a smooth curve through all the measurement points. The function of the smooth curve was denoted as f(x),so the time-weighted average of each patient can be calculated as $\bar{x}=\frac{\sum_{i=1}^{n-1}\int_{t_{i}}^{t_{i+1}}f(x)dx}{t_{n}-t_{1}}$, where n represents the amount of PaCO_2_ measurements during each patient’s ventilation time [11].

### 2.4. Statistical Analysis

Clinical data were extracted using SQL. Continuous variables were described as medians with interquartile ranges (IQRs) when not normally distributed. For group comparisons, Student’s t-test was applied to normally distributed continuous variables, while the Mann–Whitney U test was used for non-normally distributed variables. Categorical variables were compared using either the chi-square test or Fisher’s exact test, as appropriate, and results were reported as proportions.

To evaluate the effect of PaCO_2_ on survival outcomes, a restricted cubic spline (RCS) transformation was applied to model potential nonlinear relationships. An unadjusted Cox proportional hazards model was constructed, and the RCS curve was generated using the “ggrcs” package (overall *p* < 0.001; nonlinearity *p* < 0.001). HRs were estimated, and the point at which HR = 1 was identified as the reference value. The HR curve across PaCO_2_ levels was visualized using the “ggplot2” package, with confidence intervals and annotations for key points. Based on the HR = 1 reference threshold, patients were classified into three groups: hypocapnia, normocapnia, and hypercapnia. Kaplan–Meier survival curves were constructed for each group, and intergroup survival differences were evaluated using the log-rank test. Using cohort data from the merged MIMIC-IV and eICU-CRD databases as an external validation cohort, we applied the Cox proportional hazards model to assess the association between different PaCO_2_ levels and 28-day all-cause mortality across patients with various complications.

All statistical analyses were performed using R software (version 4.4.3, R Development Core Team). A two-sided *p*-value < 0.05 was considered statistically significant.

## 3. Results

### 3.1. Baseline Characteristics

According to the inclusion criteria, a total of 17,630 patients with ARF were included in the analysis, comprising 10,946 from the eICU-CRD and 6684 from the MIMIC-IV database (Table 1). In the eICU-CRD cohort, the median age was 63.00 years (IQR, 52.00–73.00) in the survival group and 69.00 years (IQR, 58.00–79.00) in the non-survival group. Similarly, in the MIMIC-IV cohort, the median age was 65.00 years (IQR, 54.00–76.00) among survivors and 70.00 years (IQR, 59.00–81.00) among non-survivors. The majority of patients in both databases were of Caucasian ethnicity (75% in eICU-CRD and 57% in MIMIC-IV). No significant differences were observed in sex between survival and non-survival groups. In all subgroups, males accounted for more than 50% of the population. In comparison to the non-survival group, the survival group exhibited significantly lower values for WBC count, platelet count, TBIL, creatinine, heart rate, and respiratory rate. Conversely, survivors exhibited higher MAP, hemoglobin levels, tidal volume, and P/F ratio compared with non-survivors. Arterial blood gas parameters differed significantly: survivors exhibited higher base excess (BE), pH, and bicarbonate (HCO_3_-) levels than non-survivors (*p* < 0.001), indicating a stronger capacity to realize internal homeostasis. Severity and comorbidity indices, including the Charlson Comorbidity Index (CCI), SOFA score, and APS III score, were significantly lower in the survival group compared with the non-survival group (*p* < 0.001). Compared with non-survivors, survivors had a lower prevalence of comorbid conditions. In the eICU-CRD cohort, the incidences of myocardial infarction (5.2% vs. 6.6%, *p* = 0.01), cerebrovascular disease (7.0% vs. 13.1%, *p* < 0.001), chronic kidney disease (6.9% vs. 9.1%, *p* < 0.001), severe liver disease (1.0% vs. 3.4%, *p* < 0.001), and sepsis (23.1% vs. 32.5%, *p* < 0.001) were significantly lower in the survival group. Similarly, in the MIMIC-IV cohort, the survivors had lower rates of myocardial infarction (18.0% vs. 21.5%, *p* = 0.001), chronic kidney disease (22.2% vs. 26.5%, *p* < 0.001), and severe liver disease (6.5% vs. 13.2%, *p* < 0.001). These comorbidities may be associated with poorer prognoses among patients with ARF. No significant differences were observed between survival and non-survival groups regarding the prevalence of congestive heart failure or chronic pulmonary disease in either database. Both databases indicate that the duration of ICU hospitalization for surviving patients is significantly longer compared to that of deceased patients (*p* < 0.001). Conversely, deceased patients tended to require a longer duration of mechanical ventilation (*p* < 0.001).

For a parallel comparison, an identical baseline analysis was conducted on the internally collected cohort. In the SRRSH-NJMU and SFH-NJMU cohorts, the incidence of chronic kidney disease (18.8% vs. 30.7%, *p* = 0.009) was significantly lower in the survival group. The survival group exhibited significantly lower values for TBIL and creatinine. Arterial blood gas parameters differed significantly: Survivors exhibited higher levels of base excess (BE) (*p* < 0.001) and pH (*p* = 0.001) than non-survivors. However, there was no statistical difference in other indicators, which may be due to the small sample size (Supplementary Table S1).

### 3.2. Nonlinear Impact of PaCO_2_ on Hazard Ratio (HR)

**Figure 2:**
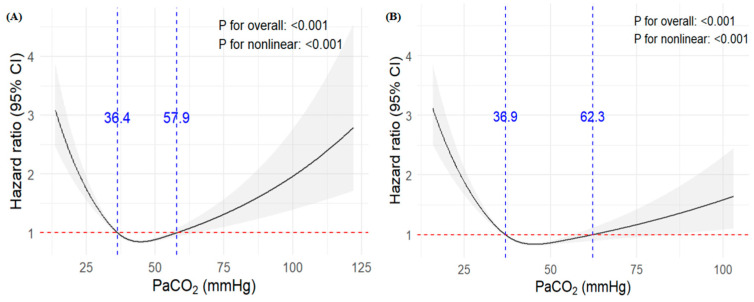
Restricted cubic spline regression analysis of PaCO_2_ with all-cause mortality. Restricted cubic spline regression analysis of PaCO_2_ and all-cause mortality in eICU-CRD (**A**) and MIMIC-IV (**B**) datasets.

In the eICU-CRD cohort, a U-shaped relationship was observed between PaCO_2_ and ICU mortality in patients with ARF, by applying an RCS in conjunction with a Cox proportional hazards model. This suggests that both excessively high and low PaCO_2_ levels are associated with increased HRs for mortality. Two inflection points corresponding to HR = 1 were identified at PaCO_2_ levels of 36.4 mmHg and 57.9 mmHg, which served as reference thresholds. The shaded region in Figure 2A represents the 95% confidence interval. Within the range of 36.4 to 57.9 mmHg, the RCS curve remained relatively flat, reflecting narrower confidence intervals and a greater statistical stability. In contrast, a PaCO_2_ value below 36.4 mmHg or above 57.9 mmHg was associated with a marked increase in HR, indicating a significantly higher risk of ICU mortality. A similar U-shaped relationship was observed in the MIMIC-IV cohort (Figure 2B). As shown in the eICU-CRD RCS analysis, patients were stratified into three groups based on these thresholds: hypocapnia (PaCO_2_ < 36.4 mmHg), normocapnia (36.4 mmHg ≤ PaCO_2_ ≤ 57.9 mmHg), and hypercapnia (PaCO_2_ > 57.9 mmHg).

### 3.3. Unadjusted Survival Analysis by PaCO_2_ Levels

**Figure 3:**
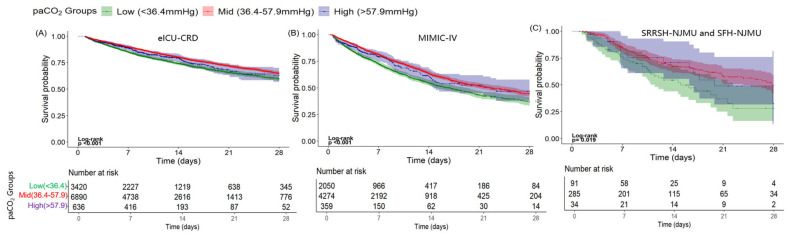
Kaplan–Meier survival analysis curves of the three cohorts. Kaplan–Meier curves showing the cumulative probability of all-cause mortality within 28 days for the Medical Information Mart for eICU-CRD cohort (**A**), the MIMIC-IV cohort (**B**), and the cohort from two university-affiliated hospitals (**C**).

Kaplan–Meier survival curves were generated to evaluate the 28-day in-hospital mortality across the three PaCO_2_ groups, revealing significant differences in survival probability. In the eICU-CRD cohort, the patients within the intermediate PaCO_2_ range (36.4–57.9 mmHg) exhibited the highest survival rate, whereas both the low PaCO_2_ group (<36.4 mmHg) and the high PaCO_2_ group (>57.9 mmHg) demonstrated substantially lower survival probabilities. The log-rank test showed statistically significant differences among the groups (*p* < 0.001), as illustrated in Figure 3A. This survival pattern was consistent across external datasets, including the MIMIC-IV cohort and the validation cohorts from Sir Run Run Hospital (SRRH) and the Second Affiliated Hospital of Nanjing Medical University (SFH), as shown in Figure 3B,C.

### 3.4. Adjusted Survival Analysis Considering Complications

**Figure 4:**
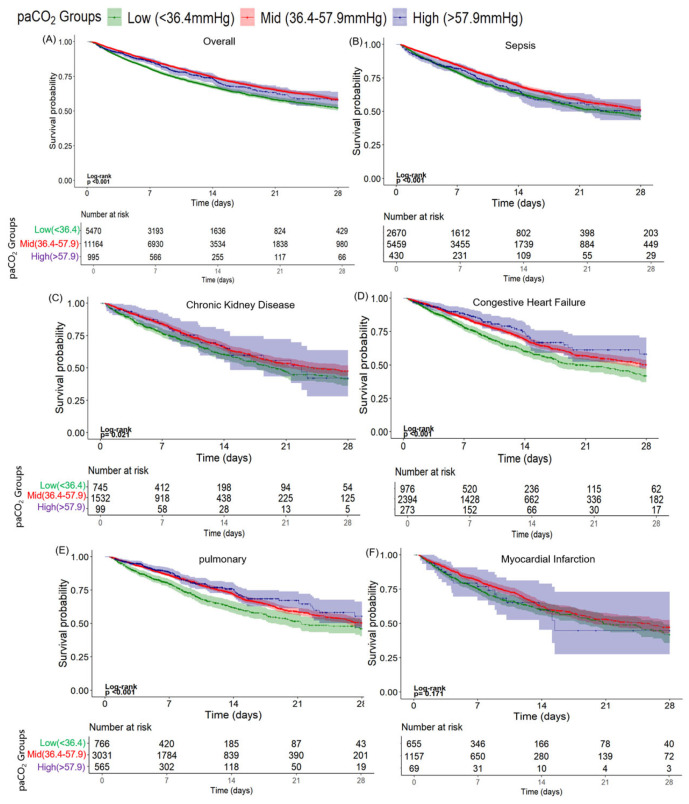
Merging the eICU-CRD and MIMIC-IV datasets. The cumulative probability of 28-day all-cause mortality for patients with ARF combined with different complications as follows: (**A**) overall, (**B**) sepsis, (**C**) chronic kidney disease, (**D**) congestive heart failure, (**E**) pulmonary disease, (**F**) myocardial infarction.

To further examine the prognostic value of PaCO_2_ levels in the context of comorbid conditions, the MIMIC-IV and eICU-CRD databases were combined to form an external validation cohort. Patients were stratified according to the presence of specific organ-related complications, and Kaplan–Meier survival curves were generated for each subgroup (Figure 4). A Cox proportional hazards regression model was applied, using the normocapnia group as the reference. The relative mortality risks associated with hypocapnia and hypercapnia were assessed across different comorbidity subgroups (Table 2). Among patients with sepsis, the PaCO_2_ level was significantly associated with the mortality risk (log-rank test, *p* < 0.001; Figure 4B). Compared with the normocapnia group, the HR for the hypocapnia group increased by approximately 25.5% (HR 1.255; 95% CI: 1.156–1.362). In the hypercapnia group, the mortality risk increased by approximately 18.9% (HR 1.189; 95% CI: 0.991–1.426), approaching the conventional threshold for statistical significance (*p* = 0.05).

A similar pattern was observed in the chronic kidney disease (CKD) subgroup (log-rank test, *p* = 0.021; Figure 4C). In comparison with normocapnic patients, those with hypocapnia had a 27.05% higher mortality risk (HR 1.271; 95% CI: 1.092–1.478). The hypercapnia group showed a 6.59% but not significant increase in mortality risk (HR 1.066; 95% CI: 0.737–1.542). Among patients with congestive heart failure (CHF), the PaCO_2_ level was significantly associated with the mortality risk (log-rank test, *p* < 0.001; Figure 4D). Compared with the normocapnia group, the patients in the low PaCO_2_ group exhibited a 36.76% increase in mortality risk (HR 1.368; 95% CI: 1.196–1.564). In contrast, the high PaCO_2_ group showed a 16.21% reduction in mortality risk (HR 0.838; 95% CI: 0.636–1.104), although this decrease was not statistically significant. In the subgroup with pulmonary complications, the mortality risk varied across PaCO_2_ stratifications (log-rank test, *p* < 0.001; Figure 4E). Compared with the normocapnia group, the low PaCO_2_ group had a 39.05% increased mortality risk (HR 1.391; 95% CI: 1.203–1.607), while the high PaCO_2_ group had an 11.08% decrease in risk that was not significant (HR 0.889; 95% CI: 0.727–1.088). In contrast, no significant association was found between PaCO_2_ level and mortality risk in the myocardial infarction (MI) subgroup (log-rank test, *p* = 0.171; Figure 4F). In this subgroup, the mortality risk in the low PaCO_2_ group was elevated by 20.4% compared to the normocapnia group (HR 1.204; 95% CI: 1.019–1.423), while the high PaCO_2_ group showed a 20.7% reduction in risk (HR 0.793; 95% CI: 0.769–1.894), although the latter association was not statistically significant.

After stratification by etiology, except for COPD, the association direction between PaCO_2_ and prognosis was consistent for ARDS, severe pneumonia, and cerebrovascular diseases, supporting 36.4–57.9 mmHg as a safe target range for acute respiratory failure patients on mechanical ventilation (Supplementary Figure S1).

## 4. Discussion

Acute respiratory failure (ARF) is a critical and complex clinical syndrome that is characterized by the respiratory system’s inability to maintain adequate gas exchange, which ultimately leads to severe hypoxemia or hypercapnia, conditions that can be life-threatening [12]. This syndrome poses a significant burden not only on the patients who suffer from it, enduring high rates of morbidity and mortality, but also on healthcare systems that are increasingly challenged to manage the rising demands for intensive care resources and specialized treatments [13]. The high incidence of ARF, particularly as a result of conditions such as pneumonia, sepsis, and acute respiratory distress syndrome (ARDS), underscores the urgent need for effective diagnostic and therapeutic strategies that can be implemented swiftly and efficiently [14]. Current management approaches primarily involve the use of mechanical ventilation and various pharmacological interventions; however, these methods have demonstrated limited efficacy in improving long-term survival rates and enhancing overall patient outcomes, indicating a pressing need for innovative solutions and comprehensive care strategies in the treatment of this serious condition [15].

In this multicenter retrospective study utilizing the eICU-CRD and MIMIC-IV databases, we investigated the association between PaCO_2_ and survival outcomes in patients with ARF. Our findings supported that both low and high extremes of PaCO_2_ are associated with an increased mortality risk [16,17]. Furthermore, we identified a PaCO_2_ range associated with a more favorable prognosis. This finding may inform future clinical research and generates a hypothesis for optimizing care strategies in patients with ARF [18,19].

In the subgroup analysis of comorbidities, the ARF patients complicated by sepsis or CKD had the lowest mortality risk in the normocapnia group, while both hypocapnia and hypercapnia were associated with a higher risk. Interestingly, in patients with coexisting pulmonary disease or chronic CHF, hypercapnia was linked to a lower mortality compared to normocapnia. Previous studies have similarly suggested potential cardioprotective effects of hypercapnia [20], possibly through stimulating brain natriuretic peptide (BNP) secretion [21]. Several studies have also proposed that mild to moderate hypercapnia may mitigate the harmful effects of elevated respiratory rate [22], improve ventilation-perfusion matching, and reduce the expression of inflammatory cytokines [23]. In patients experiencing acute exacerbations of chronic obstructive pulmonary disease (COPD), chronic CO_2_ retention is common due to long-term ventilatory limitation [24]. These patients often develop compensated respiratory acidosis and may tolerate higher baseline PaCO_2_ levels without notable adverse effects, in contrast to acutely ill individuals without prior CO_2_ retention [25]. Similar to patients with acute respiratory distress syndrome (ARDS), permissive hypercapnia is often adopted in acute COPD exacerbations to avoid complications such as barotrauma and dynamic lung overdistension from aggressive ventilatory support [26].

We observed that non-survivors had a significantly longer duration of mechanical ventilation (MV) in both cohorts. This counterintuitive finding echoes previous reports [27], which can be explained by reverse causality. Patients who died early did not have sufficient time to accumulate days of ventilation. In contrast, those who survived continued to receive mechanical ventilation, thereby increasing their length of ICU stay. Other factors include refractory hypoxemia, events related to mechanical ventilation, and delayed weaning from life-sustaining treatment. These factors prolonged the duration of mechanical ventilation and simultaneously increased mortality. Thus, the duration of mechanical ventilation seems to serve as a proxy for disease severity rather than a direct causal pathway leading to death.

This study addresses a critical gap in understanding ARF by examining the implications of varying PaCO_2_ levels on patient outcomes in a large, heterogeneous patient population. Although previous studies have established a link between abnormal PaCO_2_ levels and adverse outcomes in ARF, the distinctive contribution of the present study lies in its comprehensive analysis of extremely low and high PaCO_2_ levels using large-scale, multicenter databases. This approach enhances the external validity of the findings and provides novel insights into how these extreme values are associated with mortality risk. The multicenter design contrasts with prior investigations constrained by limited sample sizes or single-center cohorts, underscoring the value of broad, population-level analyses across diverse clinical settings [28,29]. Importantly, this is the first study to clearly demonstrate a U-shaped relationship between PaCO_2_ level and ICU mortality.

The findings of this study carry potential clinical implications. This study demonstrates that maintaining PaCO_2_ within an optimal range is crucial for improving the survival in patients with ARF. Specifically, we identified PaCO_2_ thresholds associated with a higher mortality risk, which may help clinicians refine mechanical ventilation strategies and more effectively monitor the respiratory status of critically ill patients. The upper limit of our range (57.9 mmHg) significantly exceeds the typical thresholds of concern in many clinical settings. This finding offers retrospective observational support for the safety range suggested by landmark trials on lung-protective ventilation. The 2000 ARMA trial demonstrated a mortality reduction with low tidal volume ventilation; in that study, PaCO_2_ levels in the intervention group frequently exceeded 50 mmHg, and a predefined protocol for managing respiratory acidosis triggered only when pH fell below 7.30 [30]. Our data indicate that no increased mortality risk was observed up to approximately 58 mmHg within this range, which aligns with the safety profile observed in that pivotal trial.

Furthermore, a post hoc analysis of the LUNG SAFE cohort specifically investigating hypercapnia found that mild to moderate hypercapnia, defined as PaCO_2_ levels up to 55 mmHg on the first day of ARDS, was not associated with increased mortality. This finding further reinforces the view that clinically tolerable hypercapnia includes higher PaCO_2_ values than traditionally assumed [31]. The 2023 ESICM Adult ARDS Guidelines do not set an upper limit for PaCO_2_; instead, they emphasize that lung-protective ventilation can continue as long as pH is ≥7.20 and hemodynamic stability is maintained [32]. Similarly, a recent multicenter randomized controlled trial (RCT) in ventilated neonates targeting PaCO_2_ levels of 60–75 mmHg showed no increase in intraventricular hemorrhage or mortality [33]. These results are consistent with current clinical guidelines emphasizing individualized patient management to optimize outcomes [34]. Accordingly, our findings support the incorporation of these PaCO_2_-based thresholds into routine clinical protocols aimed at reducing ARF-related mortality.

We still acknowledge the inherent limitations of this study. A retrospective design inherently carries the risk of bias, and potential confounding variables may not have been fully accounted for in our models. Additionally, use of only database-derived data may have restricted the range of clinical variables; we could only rely on arterial blood gas values extracted from electronic health records and had no access to clinicians’ real-time therapeutic intent, meaning we could not determine whether elevated carbon dioxide levels were intentional or not. Specifically, our database could not differentiate unintentional hypercapnia from intentional permissive hypercapnia, potentially affecting the accuracy of outcome evaluation. Future research should focus on prospective studies that incorporate a wider range of clinical variables or biomarker analyses to provide a more comprehensive understanding of ARF and its predictors [35,36]. Although this study excluded a large number of cases due to strict inclusion and exclusion criteria, these exclusions are related to data quality or confounding factors. This approach theoretically reduces the impact of selection bias on the main associations. While our findings highlight the association between PaCO_2_ level and mortality, further research is needed to explore the mechanisms underlying this relationship, in order to inform the development of targeted therapeutic strategies and improve the prognosis of ARF patients.

## 5. Conclusions

In this retrospective analysis, we observed an association between arterial PaCO_2_ levels within a specific range and increased survival rates. This was specifically noted in patients with acute respiratory failure on mechanical ventilation. Although this association remains significant after adjusting for available confounding factors, unmeasured factors such as treatment response, undiagnosed conditions or safety events may influence PaCO_2_ levels and outcomes. Our findings suggest a hypothesis that targeted management of PaCO_2_ may improve survival rates, but its validation in prospective studies is needed before clinical implementation.

## Figures and Tables

**Table 1:** Baseline characteristics of patients from eICU-CRD and MIMIC-IV cohort.

	eICU-CRD			MIMIC-IV		
	Survival (N = 8626)	Death (N = 2320)	*p*-Value	Survival (N = 4631)	Death (N = 2052)	*p*-Value
**Baseline variables**						
Age	63.00 [52.00, 73.00]	69.00 [58.00, 79.00]	<0.001	65.00 [54.00, 76.00]	70.00 [59.00, 81.00]	<0.001
Gender, n (%)			0.593			0.427
Female	3874 (44.1)	1057(45.6)		1961 (42.3)	891 (43.4)	
Male	4752 (55.9)	1263 (54.4)		2670 (57.7)	1161 (56.6)	
Ethnicity, n (%)			0.102			<0.001
African American	1071 (12.4)	244 (10.5)		488 (10.5)	178 ( 8.7)	
Hispanic/Native American	538 ( 6.2)	151 (6.5)		146 ( 3.2)	69 ( 3.4)	
Caucasian	6454 (74.8)	1708 (76.7)		2964 (64.0)	1216 (59.3)	
Asian	130 (1.5)	28 ( 1.2)		127 ( 2.7)	53 ( 2.6)	
Other/Unknown	433 ( 5.0)	117 ( 5.0)		906 (19.6)	536 (26.1)	
BMI	27.93 [23.63, 33.73]	27.00 [22.97, 32.95]	<0.001	28.43 [24.32, 34.04]	27.22 [23.25, 32.50]	<0.001
**Comorbidities, n (%)**						
Myocardial infarction	8175 (94.8)	2166 (93.4)	0.010	3797 (82.0)	1610 (78.5)	0.001
Congestive heart failure	7605 (88.2)	2042 (88.0)	0.875	3016 (65.1)	1323 (64.5)	0.626
Cerebrovascular disease	8020 (93.0)	2016 (86.9)	<0.001	4089 (88.3)	1785 (87.0)	0.141
Pulmonary disease	6850 (79.4)	1836 (79.1)	0.795	3143 (67.9)	1438 (70.1)	0.077
Chronic kidney disease	8031 (93.1)	2109 (90.9)	<0.001	3604 (77.8)	1509 (73.5)	<0.001
Sever liver severe	8541 (99.0)	2241 (96.6)	<0.001	4331 (93.5)	1781 (86.8)	<0.001
Sepsis	6635 (76.9)	1565 (67.5)	<0.001	3989 (86.1)	1824 (88.9)	0.002
**Vital signs**						
Heart rate (b/min)	85.95 [77.32, 95.29]	90.82 [80.75, 101.60]	<0.001	84.62 [75.16, 95.23]	89.64 [78.50, 102.24]	<0.001
MAP	84.75 [78.73, 91.45]	78.86 [72.73, 86.13]	<0.001	77.87 [72.91, 84.43]	74.11 [69.24, 80.09]	<0.001
Respiratory rate (b/min)	19.22 [17.38, 21.48]	20.89 [18.24, 23.80]	<0.001	19.45 [17.19, 21.97]	20.96 [18.24, 24.41]	<0.001
**Laboratory parameters**						
Total Bilirubin (mg/dL)	0.70 [0.43, 1.10]	0.90 [0.60, 1.70]	<0.001	0.73 [0.40, 1.77]	0.96 [0.50, 2.99]	<0.001
Hemoglobin (g/dL)	12.30 [10.70, 13.90]	11.90 [10.40, 13.70]	<0.001	9.20 [8.00, 10.70]	8.90 [7.70, 10.40]	<0.001
WBC (K/mcL)	15.90 [11.80, 21.20]	18.80 [13.59, 25.50]	<0.001	13.80 [10.10, 18.85]	16.10 [11.10, 22.60]	<0.001
Platelets (K/mcL)	262.00 [191.00, 363.00]	288.00 [220.75, 368.25]	<0.001	157.00 [103.00, 218.50]	135.00 [68.00, 203.00]	<0.001
Scr (mg/dL)	1.21 [0.86, 2.21]	1.80 [1.09, 3.40]	<0.001	0.70 [0.50, 1.00]	0.90 [0.60, 1.10]	<0.001
PH	7.43 [7.38, 7.47]	7.41 [7.35, 7.47]	<0.001	7.33 [7.26, 7.39]	7.27 [7.17, 7.36]	<0.001
PaCO_2_ (mmHg)	40.00 [35.52, 45.78]	39.06 [33.80, 45.29]	<0.001	40.02 [36.00, 45.69]	38.81 [34.09, 44.59]	<0.001
BE (mEq/L)	0.60 [−2.60, 4.00]	−1.00 [−4.80, 3.10]	<0.001	−0.50 [−3.30, 1.83]	−3.17 [−7.34, 0.00]	<0.001
HCO_3_^−^ (mmol/L)	25.00 [22.00, 28.50]	24.00 [20.50, 27.60]	<0.001	25.00 [22.00, 28.08]	22.40 [18.82, 25.75]	<0.001
**Ventilator parameters**						
RF (b/min)	19.00 [16.00, 22.00]	21.00 [18.00, 25.00]	<0.001	20.00 [16.00, 24.00]	22.00 [18.00, 28.00]	<0.001
Tidal volume	492.00 [430.00, 515.00]	468.00 [410.00, 500.00]	<0.001	480.00 [430.00, 500.00]	450.00 [400.00, 500.00]	<0.001
PEEP	5.00 [5.00, 6.00]	5.00 [5.00, 8.00]	<0.001	9.00 [5.60, 12.00]	10.00 [6.00, 13.53]	<0.001
PaO_2_/FiO_2_ ratio	227.27 [192.31, 250.00]	200.00 [150.83, 238.10]	<0.001	99.34 [97.12, 192.77]	98.42 [95.85, 163.25]	<0.001
**Score system**						
SOFA	6.00 [5.00, 9.00]	10.00 [7.00, 13.00]	<0.001	7.00 [4.00, 9.00]	9.00 [6.00, 13.00]	<0.001
APSIII	60.00 [44.00, 78.00]	78.00 [58.00, 101.00]	<0.001	51.00 [38.00, 66.00]	70.50 [52.75, 91.00]	<0.001
Charlson Index	1.00 [0.00, 2.00]	2.00 [0.00, 3.00]	<0.001	5.00 [3.00, 7.00]	6.00 [4.00, 8.00]	<0.001
**Length of stay**						
ICU length of stay, day	11.51 [6.48, 19.97]	7.09 [3.46, 12.72]	<0.001	7.47 [3.93, 13.73]	5.39 [2.50, 10.40]	<0.001
MV length of time, day	2.73 [1.16, 6.90]	3.60 [1.67, 7.14]	<0.001	1.33 [0.67, 2.75]	1.62 [0.75, 3.54]	<0.001

**Table 2:** Cox proportional hazards model for 28-day all-cause mortality.

Character	Normal	Hypocapnia		Hypercapnia	
	HR (95% Cl)	HR (95% Cl)	*p*-Value	HR (95% Cl)	*p*-Value
Overall	1	1.347 (1.265–1.434)	<0.001	1.103 (0.962–1.264)	0.160
Sepsis	1	1.255 (1.156–1.362)	<0.001	1.189 (0.991–1.426)	0.063
Chronic Kidney Disease	1	1.270 (1.092–1.478)	0.002	1.066 (0.737–1.542)	0.735
Congestive Heart Failure	1	1.368 (1.196–1.546)	<0.001	0.838 (0.636–1.104)	0.208
Pulmonary Disease	1	1.391 (1.203–1.607)	<0.001	0.889 (0.727–1.088)	0.254
Myocardial Infarction	1	1.204 (1.019–1.423)	0.029	1.207 (0.769–1.894)	0.415

## Data Availability

The data used in the present study were obtained from the MIMIC-IV and eICU-CRD database (version 2.0), which requires credential access. Researchers may obtain the dataset by applying through PhysioNet and completing the CITI training program. Data will be made available on reasonable request.

## Supplementary Materials

The following supporting information can be downloaded at: https://www.mdpi.com/article/10.3390/diagnostics16030489/s1, Table S1: Baseline characteristics of patients from SRRSH-NJMU and SFH-NJMU cohort. Figure S1: ARF etiological subgroup analysis.

## Abbreviations

PaCO2	Partial pressure of carbon dioxide
MIMIC-IV	Medical Information Mart for Intensive Care IV
eICU-CRD	eICU Collaborative Research Database
RCS	Restricted cubic spline
ICU	Intensive care unit
BMI	Body mass index
MAP	Mean arterial pressure
WBC	White blood cell count
Scr	Serum creatinine
pH	Potential of hydrogen
BE	Base excess
RF	Respiratory frequency
PEEP	Positive end-expiratory pressure
SOFA	Sequential organ failure assessment
APSIII	Acute Physiology Score III
ARDS	Acute respiratory distress syndrome
COPD	Chronic obstructive pulmonary disease
