# Supplementary material for: Association of Arterial PaCO2 with the Survival of Mechanically Ventilated Patients with Acute Respiratory Failure: A Multicenter Retrospective Cohort Study
Source: Diagnostics (Basel). 2026 Feb 5;16(3):489. doi: 10.3390/diagnostics16030489 (PMC12896418; doi:10.3390/diagnostics16030489)
Supplement: Supplementary file 1 [file diagnostics-16-00489-s001.zip › diagnostics-4035469-supplementary.pdf]

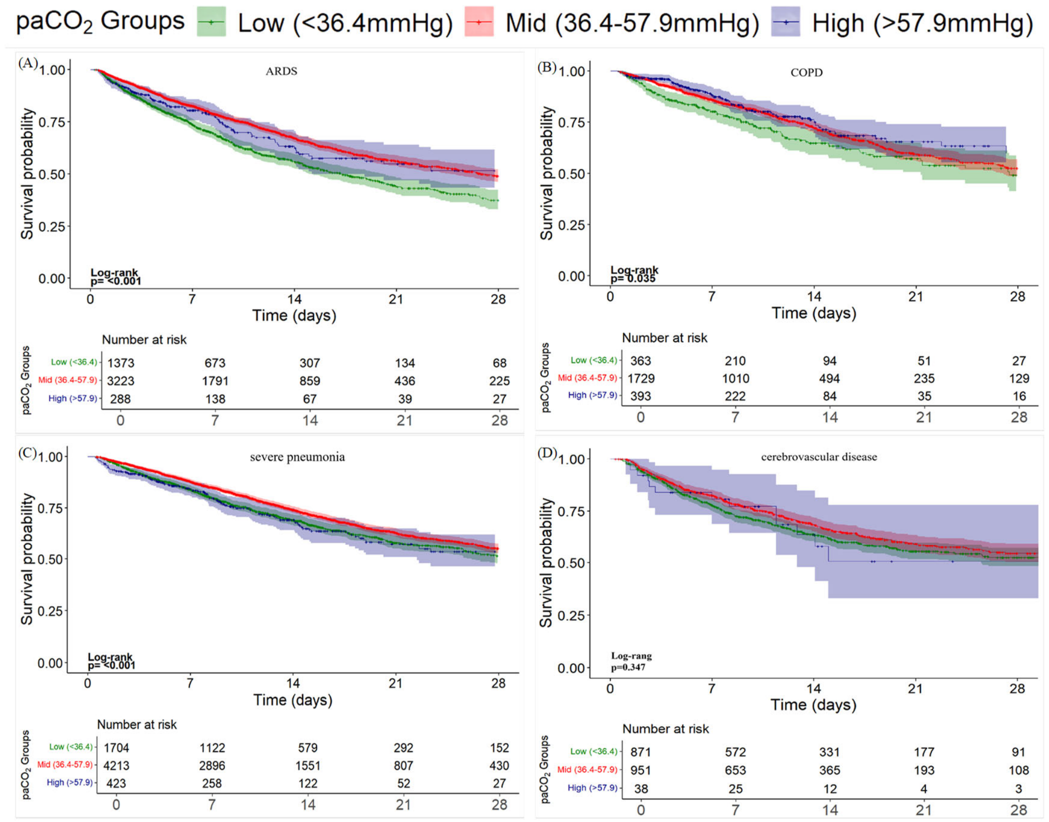

Supplementary Figure S1: Merge the eICU-CRD and MIMIC-IV datasets. Cumulative probability of 28-day all-cause mortality in patients with 4 etiologies of ARF as follows: **(A)** ARDS, **(B)** COPD, **(C)** severe pneumonia, **(D)** cerebrovascular disease.

Table S1 Baseline characteristics of patients from SRRSH-NJMU and SFH-NJMU cohort

	**Survival(N=260)**	**Death(N=150)**	**P-value**
**Baseline variables**			
Age	72.00 [60.00, 80.00]	75.00 [65.00, 83.00]	0.075
Gender,n(%)			
Female	67(25.8)	49(32.7)	0.168
Male	193 (74.2)	101(67.3)	
**Laboratory parameters**			
ALT(Units/L)	25.35 [13.10, 57.20]	30.00 [15.03, 116.62]	0.033
AST(Units/L)	32.75 [19.98, 72.38]	52.50[24.10, 183.18]	<0.001
Total Bilirubin (mg/dL)	11.70 [7.38, 19.25]	13.35 [8.80, 30.40]	0.003
NT-pro BNP (pg/ml)	770.30[230.31, 2495.18]	2278.00[556.70,7144.49]	<0.001
CK-MB(ng/ml)	6.00 [2.50, 11.70]	9.00 [3.58, 23.47]	<0.001
Scrbase(mg/dL)	81.45 [56.00, 119.25]	95.50 [69.00, 147.50]	0.005
Cr(mg/dL48h)	76.00 [53.00, 126.25]	110.50 [65.00, 187.75]	<0.001
pH	7.41 [7.35, 7.46]	7.38 [7.31, 7.43]	0.001
Lac(mmol/l)	1.70 [1.10, 2.60]	2.60 [1.40, 4.47]	<0.001
$PaCO_2$ (mmHg)	39.62 [36.73, 46.20]	40.53 [35.71, 47.76]	0.788

BE(mEq/L)	0.30 [-3.00, 4.03]	-1.75 [-5.68, 1.30]	<0.001
**ventilator parameters**			
PEEP	5.00 [5.00, 8.00]	6.00 [5.00, 8.00]	0.357
PaO2/FiO2 ratio	239.70 [164.88, 351.50]	225.06 [154.02, 367.37]	0.716
**Score system**			
SOFA-base	6.00 [4.00, 9.00]	7.00 [4.00, 11.00]	0.063
SOFA-star	7.50 [5.00, 10.00]	10.00 [7.25, 12.00]	<0.001
SOFA-48h	7.00 [5.00, 10.00]	11.00 [9.00, 13.00]	<0.001
**Comorbidities, n (%)**			
Cerebrovascular disease	135 (51.9)	81 (54.0)	0.762
Cardiovascular disease	94 (36.2)	53 (35.3)	0.952
Pulmonary	208 (80.0)	125 (83.3)	0.483
Endocrine disease	183 (70.4)	105 (70.0)	1.000
Chronic kidney disease	211 (81.2)	104 (69.3)	0.009
Digestive system disease	211 (81.2)	100 (66.7)	0.001
Sepsis	133 (51.2)	82 (54.7)	0.560
**Length of stay**			
ICU length of stay, day	13.62 [7.08, 22.39]	7.64 [5.15, 12.71]	<0.001
MV length of time, day	8.38 [4.81, 14.77]	6.68 [4.37, 11.42]	0.004

ALT Alanine aminotransferase, AST Aspartate aminotransferase,BNP Brain natriuretic peptide,NT-proBNP N-terminal pro-B-type natriuretic peptide,CK-MB Creatine kinase isoenzyme,PH Potential of hydrogen,Lac Lactic acid,BE Base excess,PEEP Positive end-expiratory pressure,SOFA-base mean the SOFA score of the first day admission,SOFA-star mean the SOFA value at the beginning of ventilation,SOFA-48h mean the SOFA value after 48 hours of ventilation
